# Prevalence, Determinants and Patterns of Multimorbidity in Primary Care: A Systematic Review of Observational Studies

**DOI:** 10.1371/journal.pone.0102149

**Published:** 2014-07-21

**Authors:** Concepció Violan, Quintí Foguet-Boreu, Gemma Flores-Mateo, Chris Salisbury, Jeanet Blom, Michael Freitag, Liam Glynn, Christiane Muth, Jose M. Valderas

**Affiliations:** 1 Central Research Unit, Institut Universitari d'Investigació en Atenció Primària Jordi Gol, Barcelona, Catalunya, Spain; 2 Universitat Autònoma de Barcelona, Bellaterra (Cerdanyola del Vallès), Spain; 3 Centre of Academic Primary Care, School of Social and Community Medicine, University of Bristol, Bristol, United Kingdom; 4 Department of Public Health and Primary Care, Leiden University Medical Center, Leiden, The Netherlands; 5 Institute of General Practice and Family Medicine, Jena University Hospital, Friedrich-Schiller-University, School of Medicine, Jena, Germany; 6 Discipline of General Practice, National University of Ireland, Galway, Ireland; 7 Institute of General Practice, Johann Wolfgang Goethe University, Frankfurt/Main, Frankfurt, Germany; 8 Health Services & Policy Research Group, School of Medicine, University of Exeter, Exeter, United Kingdom; INRCA, Italy

## Abstract

**Introduction:**

Multimorbidity is a major concern in primary care. Nevertheless, evidence of prevalence and patterns of multimorbidity, and their determinants, are scarce. The aim of this study is to systematically review studies of the prevalence, patterns and determinants of multimorbidity in primary care.

**Methods:**

Systematic review of literature published between 1961 and 2013 and indexed in Ovid (CINAHL, PsychINFO, Medline and Embase) and Web of Knowledge. Studies were selected according to eligibility criteria of addressing prevalence, determinants, and patterns of multimorbidity and using a pretested proforma in primary care. The quality and risk of bias were assessed using STROBE criteria. Two researchers assessed the eligibility of studies for inclusion (Kappa  = 0.86).

**Results:**

We identified 39 eligible publications describing studies that included a total of 70,057,611 patients in 12 countries. The number of health conditions analysed per study ranged from 5 to 335, with multimorbidity prevalence ranging from 12.9% to 95.1%. All studies observed a significant positive association between multimorbidity and age (odds ratio [OR], 1.26 to 227.46), and lower socioeconomic status (OR, 1.20 to 1.91). Positive associations with female gender and mental disorders were also observed. The most frequent patterns of multimorbidity included osteoarthritis together with cardiovascular and/or metabolic conditions.

**Conclusions:**

Well-established determinants of multimorbidity include age, lower socioeconomic status and gender. The most prevalent conditions shape the patterns of multimorbidity. However, the limitations of the current evidence base means that further and better designed studies are needed to inform policy, research and clinical practice, with the goal of improving health-related quality of life for patients with multimorbidity. Standardization of the definition and assessment of multimorbidity is essential in order to better understand this phenomenon, and is a necessary immediate step.

## Introduction

Multimorbidity − the presence of more than one health condition in an individual [1], [2] − is increasingly being recognised as the norm rather than the exception in primary care patients [3]. Multimorbidity increases the risk of premature death, hospitalizations, loss of physical functioning, depression, polypharmacy, and worsening quality of life, translating into a substantial economic burden for health systems [4]. Information on the prevalence of multimorbidity and the most frequent combinations of health conditions is essential for optimum organisation and delivery of health care [5], [6]. The identification of the key determinants of multimorbidity is a prerequisite for the development of effective strategies for the early identification of patients at risk and for the prevention of future health conditions [7].

A number of studies have examined the prevalence of multimorbidity, with methods for estimation ranging from simple counts of the number of diseases per individual to sophisticated patient classification systems for the measurement of morbidity burden and case-mix [1]. Recently published systematic reviews have tried to summarise these studies, but they are not without limitations, such as omitting information on the determinants and/or patterns of multimorbidity [8]–[10] and an exclusive focus on longitudinal studies [11]. Not least, they quickly became outdated. A number of relevant studies have been published in the last few years, and there is an urgent need to establish what is currently known about the determinants and prevalence of multimorbidity and the most frequent patterns observed in primary care.

Our aim was to systematically review and synthesise the available evidence on the prevalence, major determinants and patterns of multimorbidity in primary care in order to inform the organisation and delivery of primary care.

## Methods

We conducted a systematic review of the literature for reports of studies aimed at estimating the prevalence of multimorbidity and/or determining the predominant patterns or combinations of health conditions in primary care patients. We defined multimorbidity as “the simultaneous presence of more than one health condition in the same individual” and multimorbidity patterns as “the simultaneous presence of multiple specific health conditions in the same individual”. We considered two types of multimorbidity patterns: the most frequent combinations of specific diseases (pairs and triplets), and the groups of health conditions with the highest degree of association using the corresponding statistical analyses (cluster and factor analysis).

### Study selection

We included primary studies reporting the prevalence of multimorbidity and/or the prevalence of patterns of multimorbidity in primary care. Four exclusion criteria were applied: a) articles not reporting original research (reviews, editorials, non-research letters); b) studies that recruited patients through in non-primary care settings (hospitals, nursing homes, etc.); thereby limiting representativeness for Primary Care; c) studies that recruited patients based on specific characteristics such as the presence of any specific condition (e.g., diabetes), or any sociodemographic characteristic other than age; and d) studies using patients' self-reported diagnoses.

### Search strategy

A protocol was developed using PRISMA guidelines [12]. It is available at http://www.phc.ox.ac.uk/research/hsprg/research-projects/multimorbidity/Protocol.SR.PDPMM.1.web.txt. We used an established, structured methodology for the analysis of electronic databases, which combined a pre-defined search strategy with a valid snowball method. The latter is particularly useful when terminology has not been applied consistently in the literature [1]. We used Ovid to search PubMed-Medline (1960 to July 10, 2013), EMBASE (1980 to July 10, 2013), PsycINFO (1948 to July 10, 2013) and CINAHL. A structured search strategy combining text and MeSH terms identified relevant articles (Table S1). No language restrictions were applied.

We searched Web of Knowledge for all citing and cited articles for each eligible paper (backward and forward search). These references were included in the pool of references to be screened and were subjected to the same screening processes as those retrieved from any other database.

### Eligibility assessment and data extraction

Two researchers (CV and QFB) assessed the eligibility of studies for inclusion. Disagreements were mediated by JMV and resolved by consensus. A pilot test with a sample of studies showed high inter-rater reliability (Kappa  = 0.86).

**Figure 1:**
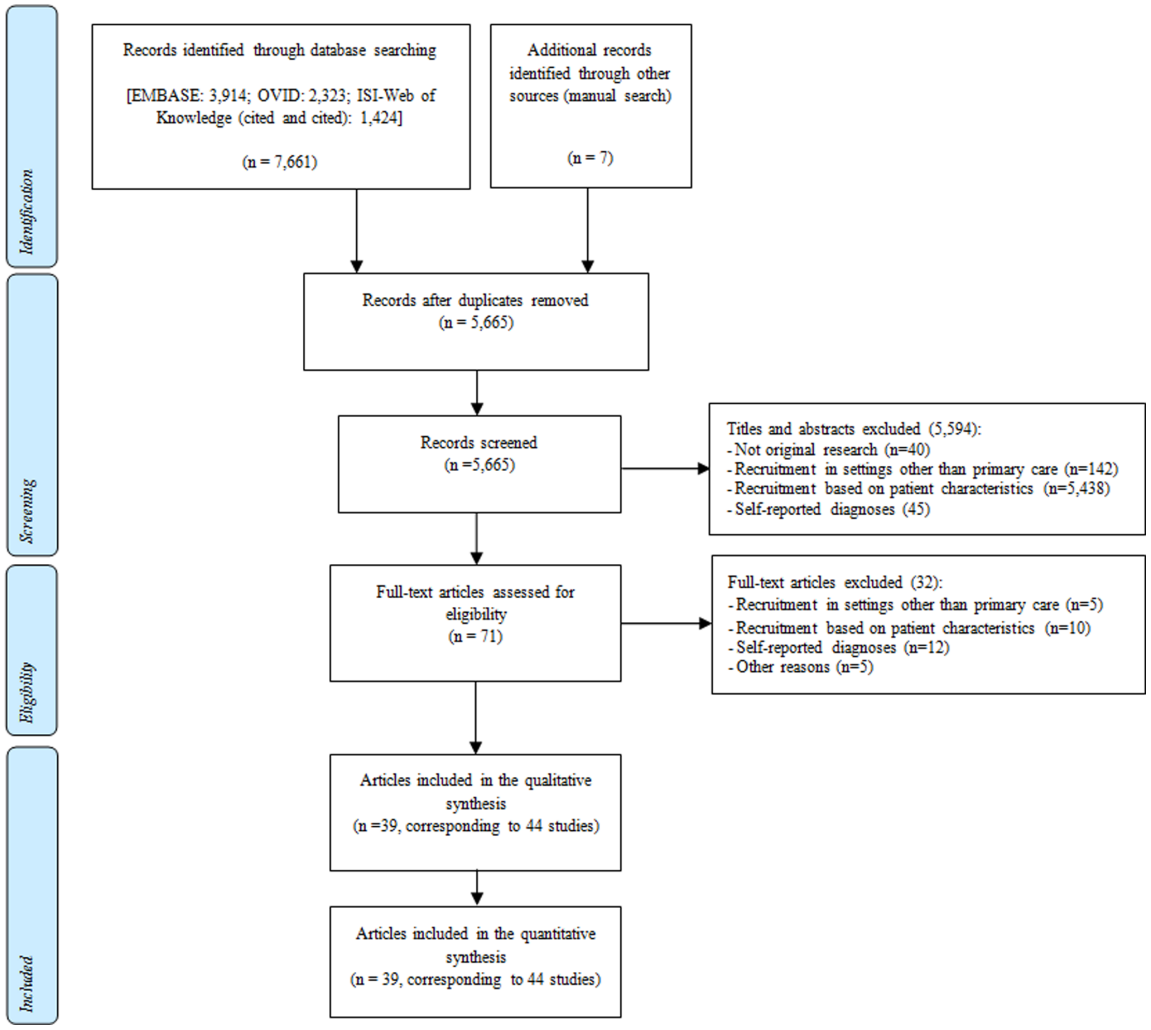
Flow chart for study identification.

Relevant information from the selected articles was extracted by all the researchers using a standardised proforma. For each article, two authors independently extracted data on the prevalence of multimorbidity and of the most frequent patterns, along with publication year, country, study design, sample size, sampling method, data source, coding system and/or method for the identification of health conditions, number of health problems considered as eligible, age range of participants, proportion of women, methods for modelling of multimorbidity patterns (where applicable), and all the variables analysed to establish the determinants of multimorbidity (Figure 1).

Two authors (CV and QFB) used the STROBE checklist for observational studies to assess the quality of each study (Table S2) [13]. We conducted a pilot test of the data extraction process to ensure high inter-rater agreement (Kappa = 0.96). Any disagreements were mediated by JMV and resolved by consensus.

### Data analysis

We report descriptive statistics for the estimates of the prevalence of multimorbidity and the associated patterns. Heterogeneity estimates were too high (*I* ^2^>90% for all analyses) to support quantitative pooling to confirm qualitative assessment based on key study characteristics (Table S2). We studied the association between prevalence estimates and selected study characteristics using non-parametric tests (Spearman correlation). We tabulated the significant multimorbidity patterns according to age and gender whenever possible. We created forest plots for the association between multimorbidity prevalence and the determinants that had been evaluated in some of the studies. If the original study did not report any standard measure of association, we calculated odds ratios (OR) based on the proportion of patients with and without multimorbidity. We used STATA version 11 throughout (STATA Corp, College Station, TX), except for odds ratios for the determinants of multimorbidity, which were calculated using EPIDAT version 3.1. [14].

## Results

The electronic and manual searches yielded 7,668 references, of which 71 were potentially relevant. These references were reviewed in full text, leading to the inclusion of 39 articles [15]–[53] corresponding to 44 studies (see Figure 1). One article included results from five different samples [43] and another reported results from two different samples [50] (Table S2).

Sample size ranged from 328 [19] to 31,313,331 [25] participants. Of the 44 studies, 33 were conducted in Europe [15]–[17], [19], [21]–[24], [26]–[30], [34]–[35], [37], [39]–[41], [43], [45]–[52], of which the majority were conducted in the Netherlands (12 studies) [29], [41], [43], [45]–[48], [51], Germany (6 studies) [24], [39], [40], [49], [50], and the United Kingdom (5 studies) [16], [17], [23], [26], [37]). Eight studies were conducted in North America, six in the United States [25], [32], [33], [42], [44], [53], and two in Canada [20], [31], and three studies were conducted in Australia [18], [36], [38].

In the 32 studies using data from health records, registers or administrative claims [16]–[17], [20]–[26], [29], [31]–[39], [41]–[47], [49]–[53], a variety of classification systems were used, including different versions of the International Classification of Primary Care (ICPC) [22], [24], [29]–[30], [34]–[35], [38], [41], [43], [45], [46]–[48], [51], the International Classification of Diseases (ICD) versions 9 and 10 [25], [27], [28], [32], [33], [39], [40], [42]–[44], [49], [50], [52], [53] and Read codes (the clinical coding system used in General Practice in the UK) [16], [17], [23], [26]. One study used a combination of ICD–9 and International Classification of Health Problems for Primary Care (ICHPPC–2) [36]. Only three studies [22], [35], [38] included all chronic health conditions and used O'Halloran criteria for chronic disease (147 codes) [54]. The remaining studies (93.2%) selected a variable number of conditions, which ranged from 5 [41] to 335 [47].

### Study quality

Quality was high in most of the studies (median score 18 out of a maximum STROBE score of 23 (range: 5–23, Table S2). The two main weaknesses were: insufficient efforts to address potential sources of bias and sparse information for each variable of interest on the number of participants with missing data.

### Prevalence of multimorbidity

**Figure 2:**
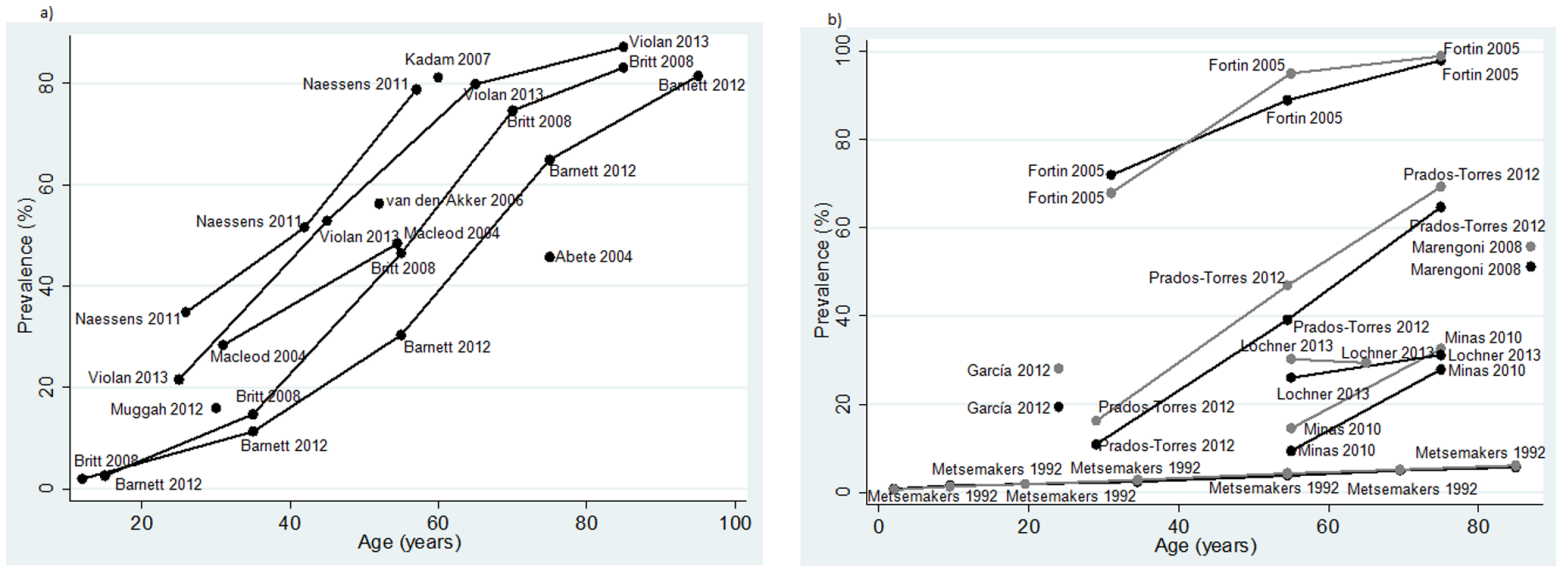
Prevalence of multimorbidity by age group: overall (a) and by sex (b).

Thirty-nine studies measured the prevalence of multimorbidity. Overall estimates ranged from 12.9% in participants aged 18 years and older [51] to 95.1% [19] in a population aged 65 years and older. With the exception of five studies [17], [31], [35], [45], [51], all estimates exceeded 20% (Figure 2). The most common design (56.8%) was cross-sectional [15], [16], [18]–[26], [29]–[31], [36], [39], [41], [43], [46], [47], [49], [52], [53] (Table S2).

The definition of multimorbidity differed between studies. In 25 studies, multimorbidity was defined as the presence of at least 2 chronic diseases in the same person [15], [16], [18], [19], [21], [22], [24], [25], [27], [28], [33], [34], [35], [37], [43], [44], [45], [47], [48], [51], [52]; in 5 studies as the presence of at least 3 chronic diseases [39], [40], [49], [50]; in 12 studies by counting the total number of medical conditions and defining groups accordingly [20], [23], [26], [30]–[32], [36], [38], [41], [42], [46], [53]; and 2 studies did not report these data [17], [29].

No association was observed between the overall prevalence of multimorbidity and any of the selected study characteristics (design, sample size, sampling, data source, coding system), except for a slight (non-significant) positive correlation with the number of health conditions considered (Spearman rho: 0.28; *p* = 0.11).

### Determinants of multimorbidity

**Figure 3:**
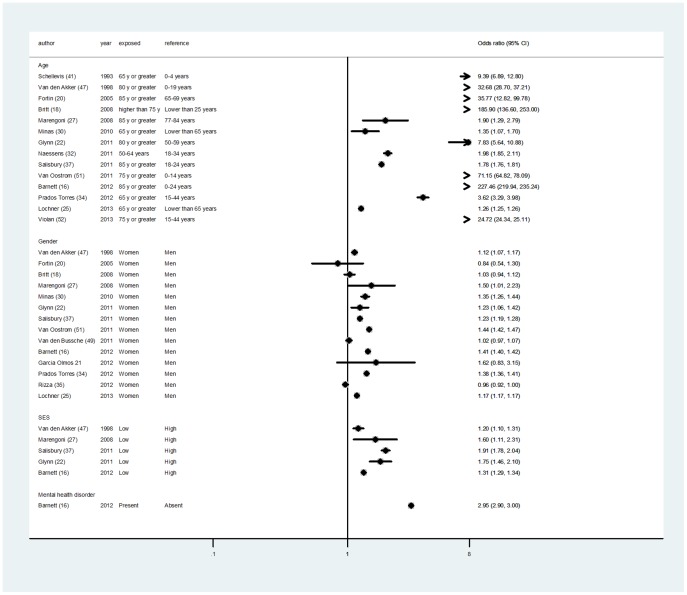
Forest plots for determinants of multimorbidity: odds ratios (ORs) and 95% CIs for age, gender, socioeconomic status (SES) and existing mental disorder.

Age was the most frequently studied determinant of multimorbidity [16], [18], [20], [22], [25], [27], [30], [32], [34], [37], [41], [47], [51], [52]. All the studies showed a significant positive association between age and prevalence of multimorbidity (Figure 3). A number of other studies confirmed this observation, although they reported data that could not be used in the forest plot [21], [26], [35], [36], [39], [44], [45], [46], [53].

Fourteen studies assessed the association between prevalence of multimorbidity and gender [47], [20], [18], [27], [30], [22], [37], [51], [49], [16], [21], [34], [35], [25] (Figure 3). Prevalence was significantly higher in women in nine studies [16], [22], [25], [27], [30], [34], [37], [47], [51] and non-significantly higher in three additional studies [18], [21], [49]. Two studies showed a slightly higher prevalence in men, but the difference was non-significant [20], [35]. In one study, these comparisons were age-adjusted [26].

Five studies examined the association between prevalence of multimorbidity and socioeconomic status, measured using a deprivation index [16], [37], health insurance coverage [22], [47] and educational level [27], [47]. Consistently, all these studies showed an inverse association between socioeconomic status and multimorbidity. The OR for comparisons of the lowest and highest socioeconomic status ranged from 1.20 (95% CI 1.10 to 1.31) [47] to 1.91 (95% CI 1.78 to 2.04) [37]. Except for two studies [16], [22], all socioeconomic comparisons were adjusted for age and gender.

One study found the presence of mental health disorders to be significantly associated with the prevalence of multimorbidity adjusted for age and gender [16]. No studies assessed the impact as determinants of multimorbidity of any specific conditions or well-established risk factors (e.g., smoking and high blood pressure) that are independently associated with the incidence of health conditions commonly present in patients with multimorbidity.

### Patterns of multimorbidity

**Figure 4:**
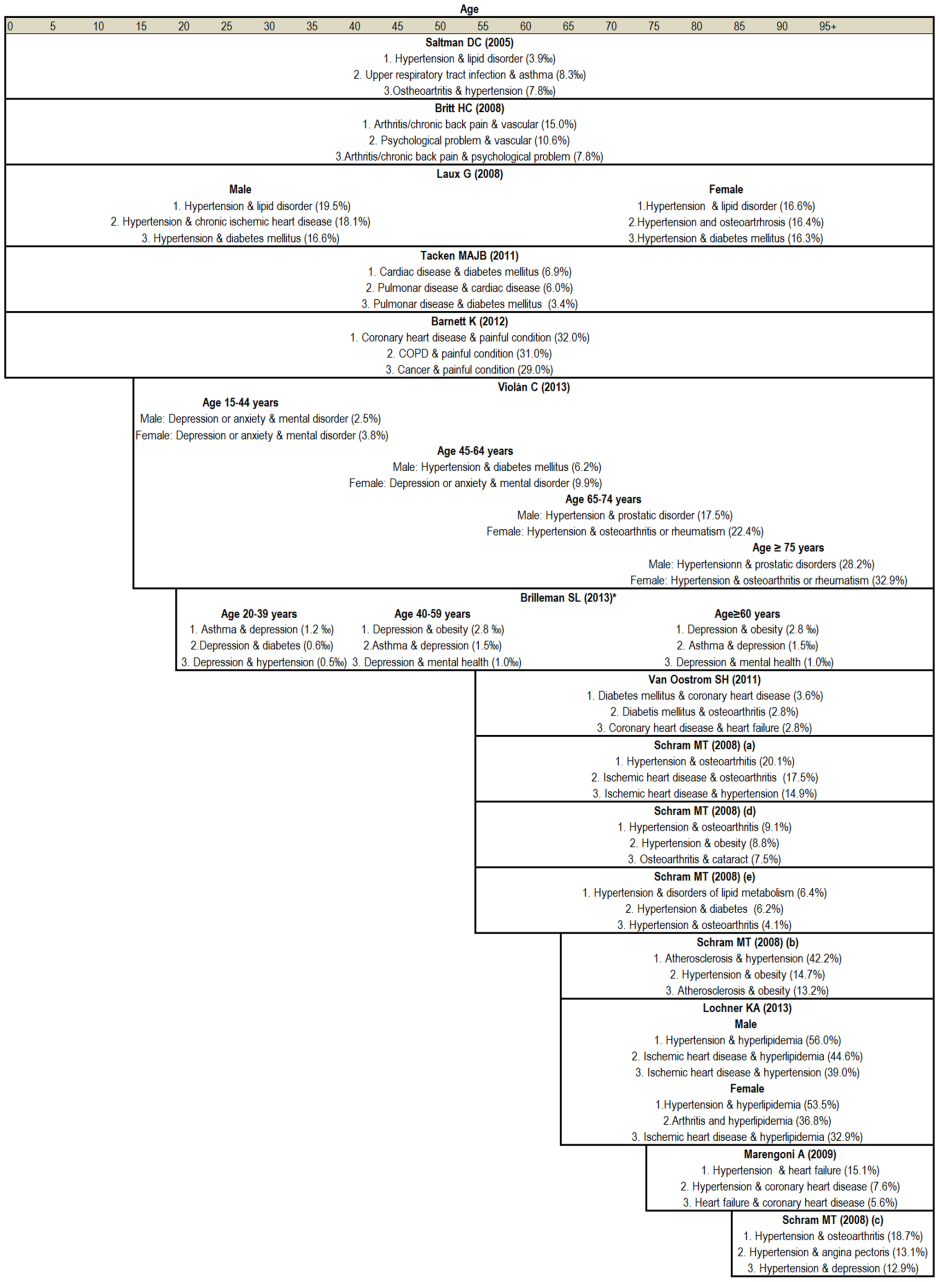
Most frequent pairs of health conditions.

Only 24 studies of 44 [16], [18], [19], [21], [24], [25], [28], [33], [34], [38]–[40], [43]–[45], [49]–[52] provided information on patterns of multimorbidity. Most of these (11 studies) focused on descriptive information pertaining to the frequency of all possible combinations of two conditions [16], [18], [24], [28], [38], [43], [51] and three studies described combinations of two and three conditions [25], [45], [52]. Hypertension and osteoarthritis was the most frequent combination, followed by different combinations of cardiovascular conditions. In general, the most frequent pairs were made up of the most frequent single conditions in each study (Figure 4). Four studies [44], [49], [50] analysed combinations of three conditions (data not shown).

As for the identification of meaningful groups of conditions, two studies used cluster analysis [19], [33], and four used factor analysis [21], [34], [39], [40] (Table 1). There was no consistent pattern across the clusters among the first set of studies. Three of the four factor analysis studies stratified their results by gender [34], [39], [40] and only one stratified both by gender and age [34]. As with cluster analysis, there was variation in the composition of the factors. However, a rather consistent picture emerged, with a number of factors being common across studies, including a factor comprising a variety of cardio-metabolic conditions (on occasion split into two factors), a factor that included anxiety and depression (on occasion associated with other psychiatric conditions), and a factor including pain (on occasion associated with anxiety and depression).

**Table 1:** Patterns of multimorbidity.

Statistical method	First Author (year)	Multimorbidity patterns
Cluster analysis	Newcomer SR (2011)	1. Chronic pain & mental health conditions (6.6%)
		2. Diabetes & obesity & mental health conditions (12.0%)
		3. Kidney disease & diabetes & obesity (14.0%).
	Formiga F (2012)	1. Auditory impairment*
		2. COPD and malignancy*
		3. Atrial fibrillation, heart failure, visual impairment, CKD, stroke, high blood pressure and diabetes mellitus*
Factor analysis	Prados-Torres A, 2012	**Male:**
		15–45 years:1. Cardio-metabolic (0.9%)
		2. Psychiatric-substance abuse (1.5%).
		45–64 years:1. Cardio-metabolic (9.2%)
		2. Mechanical-obesity-thyroidal (4.9%).
		≥65 years:1. Cardio-metabolic-obesity-thyroidal (20.4%)
		2. Mechanical-obesity-thyroidal (1.7%)
		3. Psychogeriatric. (13.5%).
		**Female:**
		15–45 years:1. Cardio-metabolic (0.4%)
		2. Mechanical-obesity-thyroidal (2.7%).
		45–64 years:1. Cardio-metabolic (4.1%)
		2. Mechanical-obesity-thyroidal (11.7%)
		3. Depressive (0.1%).
		≥65 years:1. Cardio-metabolic (33.3%)
		2. Mechanical-obesity-thyroidal (3.5%).
		3. Psychogeriatric (17.3%).
	Schäfer I (2010)	**Male:**
		1. Cardiovascular & metabolic disorders (39.0%)
		2. Anxiety/depression/somatoform disorders & pain (22.0%)
		3. Neuropsychiatric disorders (0.8%)
		**Female:**
		1. Cardiovascular & metabolic disorders (30.0%)
		2. Anxiety/depression/somatoform disorders & pain (34.0%)
		3. Neuropsychiatric disorders (6.0%),
	Schäfer I (2012)	**Male:**
		1. Cardiovascular/metabolic disorders (79.8%).2. Anxiety, depression, somatoform disorders and pain (46.0%)
		**Female:**
		1. Anxiety, depression, somatoform disorders & pain (66.4%)2. Cardiovascular/metabolic disorders (55.2%)
	Garcia L (2012)	1. Hypertension & disorders of lipid metabolism & type2 diabetes & cardiac arrhythmia*
		2. Cerebrovascular disease & ischemic heart disease & chronic renal failure & congestive heart failure*
		3. Anxiety and depression & thyroid disease & asthma & schizophrenia and affective psychoses*

*No prevalence data reported for this study.

## Discussion

This is the first systematic review to provide a comprehensive analysis of multimorbidity prevalence, determinants and patterns. There are five major findings: a) estimates of multimorbidity prevalence and the identification of specific patterns vary widely between studies (from less than 15% to more than 95%); b) there is huge variation in sample selection criteria and in the methods for estimating multimorbidity (eligible conditions, coding systems), their patterns (including also the different types of analysis: probabilistic pairs and triplets, cluster analysis, factor analysis); c) all too frequently there is lack of adherence to some fundamental principles of epidemiological studies, such as reporting by age and gender; d) multimorbidity has been consistently associated with age (the majority of individuals older than 65 years have multimorbidity), female gender, lower socioeconomic status, as well as the presence of mental health problems; and e) the most frequent combinations were those that included osteoarthritis and a cardio-metabolic cluster of conditions such as high blood pressure, diabetes, obesity and ischaemic heart disease.

Although estimates for the prevalence of a condition may vary with different recruitment and sampling methods, classification systems, and local peculiarities in coding, among other factors, we were not able to identify any statistically significant association with such characteristics of study design. Another aspect to consider is the sources of study data. Data from health records, registers and administrative claims may detect less complex problems and under report diseases. Health conditions more frequently registered in health records could be conditioned by their severity (cardiac disease and malignant tumour) or by the fact that some chronic conditions are of particular interest (such as diabetes mellitus and hypertension in many quality incentive schemes). Other conditions (such as dementia, some mental health conditions), may be under-diagnosed and underreported [52]. The lack of valid and reliable methodological standards for the identification of multimorbidity may have limited the strength of statistical analysis, affecting both the estimates of overall prevalence and the identification of patterns of multimorbidity. Differences in the number of eligible conditions ranging from 5 to 335 would clearly have a substantial impact on the observed characteristics of patients with multimorbidity.Until appropriate standards are developed and adopted by the research community, this problem may well continue to constitute one of the most significant barriers to the advancement of research in this area.

The association between multimorbidity and age has been established in almost every study on the issue and is consistent with the notion that the additional life-years constitute an additional opportunity for acquiring other chronic conditions. It seems a consistent observation that, almost regardless of the methods used, multimorbidity is the norm in those aged 65 or older. The increased frequency with which multimorbidity occurs among women demands an explanation. Although it might be in part attributed to the lack of simultaneous adjustment or stratification for age and gender in the majority of studies, those that did make both adjustments also observed an increased prevalence among women (with OR ranging from 1·12 to 1·50). It is not possible to disentangle from the current data an explanation of whether this is due to residual confounding, higher consultation rates in women leading to higher rates of diagnosis, differential numbers of gender-specific conditions in each study or indeed differences in the burden of health conditions. If the latter were truly the case, it would provide an explanation for the well-established fact that women tend to rate their health and health-related outcomes as worse than men [55].

The methods used to provide information on multimorbidity patterns were inconsistent across the studies that focussed on this research question. The information provided by the authors does not allow comparison of the methods used to establish which approach is the best.

### Strengths and weaknesses of this review

This review has a number of strengths compared to previous reviews that provide data on the prevalence of multimorbidity in primary care. We identified a much larger number of studies (39 articles, compared to 21 articles in the most extensive review by other authors [9]). The review by Marengoni et al was limited to the population aged 65 years and older, and included patients admitted to hospitals and nursing homes; for these reasons, our results cannot be compared [2]. Likewise, the number of cohort studies identified in our review is far greater than those analysed by other researchers [11]. None of these previous reviews considered the most frequent multimorbidity patterns and the associated determinants.

However, our study also has limitations. Although identification and selection bias are a common threat to validity in all systematic reviews, they are more likely in reviews of non-randomised studies, because study registration is not standard practice. Particular efforts have been devoted to reduce identification bias, as evidenced by our search strategy including several databases, enhanced with forward and backward citation mining. Estimates of prevalence, determinants and patterns in our study are limited by the methods used in the primary studies. All studies were conducted in high-income Organisation for Economic Co-operation and Development (OECD) countries. In middle- and low-income countries, however, communicable health conditions play a much larger role in the disease burden and would be expected to significantly affect prevalence, determinants and patterns of multimorbidity. We were not able to combine studies to obtain an overall estimate, due to substantial statistical and methodological heterogeneity. More generally, the design of the original studies obviously imposes limits on our ability to establish inferences, as it is particularly the case for the issue of the determinants of multimorbidity. However, the consistency of our observations on the association between multimorbidity and its determinants supports our confidence in our observations.

### Implications for clinical practice, health policy, and future research

The high prevalence of multiple conditions makes an increased focus on the routine delivery of specific multimorbidity interventions necessary. Elderly women of lower socio-economic status seem to be at the highest risk for multimorbidity, particularly in the presence of mental disorders. Although the evidence base for the management of multimorbidity appears to be thin and much more research is still needed [4], current best practice should focus on the prevention of common risk factors and an orientation of treatment towards the improvement of functional limitations. Furthermore, in light of this review, expert consensus seems to be essential to establish an operational definition of multimorbidity that facilitates comparison between different world regions. Patients with multimorbidity need coordinated and continuing care. These are core functions of Primary Care. Payment systems will need to appropriately take these roles into account and reward them accordingly (e.g., it is more complex to manage a patient with a number of different conditions than the same number of patients each with a single condition).

Research is particularly needed on the clustering of conditions in patients with multimorbidity. From an etiological perspective, it is important to understand what makes conditions tend to co-occur, with the aim of being able to prevent their development in the first place. On the other hand, from a clinical perspective with a focus on ongoing management of patients with multimorbidity, the identification of particularly frequent associations is important relevant to identifying therapeutic approaches that take comorbidity into account and allow the tailoring of care for significant strata of people with a given condition or combination of conditions. Finally, from a policy perspective, targeting both modifiable determinants of multimorbidity and common risk factors for conditions pertaining to the same cluster will be an efficient approach to preventing multimorbidity and its associated risks.

Progress will continue to be impaired by poor design of studies of multimorbidity. Until formal consensus on the best methods for the study of multimorbidity and multimorbidity patterns is developed, these studies should consider the following minimal standards: a) unrestricted eligibility of conditions rather than selected subsamples of conditions, in order to ensure replicability and comparability across studies, or less ideally, agreement on a defined list of key conditions; b) diagnoses confirmed by health professionals, using established coding systems [56]; and c) reporting of results stratified by age and gender. Research comparing different methods for the identification of beyond chance association and clustering of conditions is urgently needed. Further research is also needed on the association between multimorbidity and modifiable risk factors such as smoking and diet, and explanatory research into gender differences. Finally, we lack fundamental information about multimorbidity in lower and middle-income countries where patterns of disease will more frequently include communicable diseases, such as malaria, tuberculosis and HIV infection. A coordinated effort similar to that which resulted in the Global Burden of Disease study would be needed [57].

## Conclusions

Although multimorbidity estimates and patterns are heavily dependent on the measurement methods, there is evidence that a substantial proportion of the primary care population is affected. Well-established determinants of multimorbidity include age, gender, and lower socioeconomic status, and it has also been associated with the presence of mental disorders. However, the substantial limitations identified across the studies included in this review means that available evidence is not enough, and much more research is needed. In particular, the standardization of the definition and the assessment of multimorbidity is essential in order to better understand this phenomenon.

Clinical practice and health policy needs to orient the delivery of care to ensure that the resources match the needs of this group of complex patients.

## Supporting Information

Table S1 **Search Strategies for the Electronic Databases.**
(DOCX)

Table S2 **Studies included in the systematic review.**
(DOCX)

Checklist S1 **PRISMA Checklist.**
(DOC)
