# Supplementary material for: Prevalence, Determinants and Patterns of Multimorbidity in Primary Care: A Systematic Review of Observational Studies
Source: PLoS One. 2014 Jul 21;9(7):e102149. doi: 10.1371/journal.pone.0102149 (PMC4105594; doi:10.1371/journal.pone.0102149)
Supplement: Table S1 — Search Strategies for the Electronic Databases. (DOCX) [file pone.0102149.s001.docx]

**Table S1. Search Strategies for the Electronic Databases**

| **Database** | **Search Strategy** | **References** |
| --- | --- | --- |
| OVID  (CINAHL, PsycINFO, MEDLINE  and  EMBASE)* | ((comorbidity or comorb$ or co?morb$ or multimorb$ or multi?morb$ or polymorb$ or poly?morb$ or multipatholog$ or multi?patholog$ or polypatholog$ or poly?patholog$ or pluripatholog$ or multidiseas$ or multidiagnos$ or polydiagnos$ or multicondition$ or multidisor$ or multiproblem$ or multiple chronic conditions$ or multiple chronic diseases$).ti.) AND (prevalence.sh. or prevalence$.ti. or epidemiology$.ti. or pattern$.ti. or cluster$.ti. or number$.ti.) | 6,237 |
| Web of Knowledge | Snow balling: references cited in the eligible papers (forward), and references citing the eligible papers (backward) | 1,424 |

* Except for EMBASE inception period to 2012, slightly modified search strategy to accommodate differences in terminology
