# Supplementary material for: Prevalence, Determinants and Patterns of Multimorbidity in Primary Care: A Systematic Review of Observational Studies
Source: PLoS One. 2014 Jul 21;9(7):e102149. doi: 10.1371/journal.pone.0102149 (PMC4105594; doi:10.1371/journal.pone.0102149)
Supplement: Table S2 — Studies included in the systematic review. (DOCX) [file pone.0102149.s002.docx]

**Table S2. Studies included in the systematic review**

MM: multimorbidity; NR: not reported. CPC International Classification of Primary Care; ICHPPC-2: International Classification of Health Problems in Primary Care; ICD-9-CM: International Classification; EDCs: Expanded Diagnostic Clusters; ADGs: Ambulatory Diagnostic Groups.

* Multimorbidity definition-measurement considered in the articles: a) ≥ 2, the coexistence of two or more diseases in the same individual; b)≥ 3, the coexistence of three or more diseases in the same individual; and b) Count, by counting the number of diseases.
